# Effects of Cyclic Tensile Strain on Chondrocyte Metabolism: A Systematic Review

**DOI:** 10.1371/journal.pone.0119816

**Published:** 2015-03-30

**Authors:** Judith Bleuel, Frank Zaucke, Gert-Peter Brüggemann, Anja Niehoff

**Affiliations:** 1 Institute of Biomechanics and Orthopaedics, German Sport University Cologne, Köln, Germany; 2 Center for Biochemistry, Medical Faculty, University of Cologne, Köln, Germany; 3 Cologne Center for Musculoskeletal Biomechanics, Medical Faculty, University of Cologne, Köln, Germany; University of Umea, SWEDEN

## Abstract

Chondrocytes reorganize the extracellular matrix of articular cartilage in response to externally applied loads. Thereby, different loading characteristics lead to different biological responses. Despite of active research in this area, it is still unclear which parts of the extracellular matrix adapt in what ways, and how specific loading characteristics affect matrix changes. This review focuses on the influence of cyclic tensile strain on chondrocyte metabolism *in vitro*. It also aimed to identify anabolic or catabolic chondrocyte responses to different loading protocols. The key findings show that loading cells up to 3% strain, 0.17 Hz, and 2 h, resulted in weak or no biological responses. Loading between 3–10% strain, 0.17–0.5 Hz, and 2–12 h led to anabolic responses; and above 10% strain, 0.5 Hz, and 12 h catabolic events predominated. However, this review also discusses that various other factors are involved in the remodeling of the extracellular matrix in response to loading, and that parameters like an inflammatory environment might influence the biological response.

## Introduction

**Fig 1:**
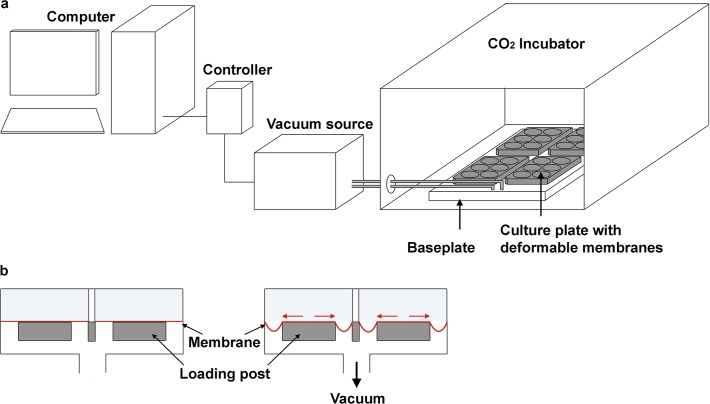
Schematic view of a method to stretch cell in vitro. **a**: Experimental setup of a cell stretching device. The loading protocol is transferred from the computer to a vacuum pump by a control unit. The vacuum source is connected to a baseplate within an incubator, where the cell culture plates with deformable membranes are inserted hermetically sealed. **b**: Cross sectional view of the cell culture plates and the deformable membranes *(in red)* without *(left)* and with *(right)* applied vacuum. The picture on the *right* demonstrates the stretching of the membranes over loading posts under the influence of the vacuum. The cells are attached on the membranes and are thereby exposed to tensile strain. Inter alia, the parameters strain magnitude, frequency and loading duration can be configured.

Articular cartilage has the function to transmit forces across joints, to minimize peak stresses and to provide nearly frictionless gliding of the articular surfaces. Consequently, the chondrocytes are permanently exposed to a combination of different forces, like compression, tension, and shear. These mechanical signals acting on articular cartilage are critical regulators of tissue adaptation, structure, and function [1]. It is well accepted that different kinds of mechanical loading lead to different biological responses [2,3]. However, distinct anabolic or catabolic loading protocols, and the subsequent processes of adaptation remain to be elucidated. The effects of compression and shear forces on chondrocytes in three-dimensional *in vivo* and *in vitro* experiments have been investigated in details, and have already been summarized in several reviews [4–7]. However, cartilage compression exposes the chondrocyte to compressive forces, to osmotic pressure, to fluid flows and also to tensile forces [8–12]. It is difficult to eliminate the effects of other physical factors with *in situ* or *in vivo* investigations. Therefore, besides those experiments, two-dimensional *in vitro* cell loading experiments were carried out [13,14] (Fig. 1). With these, cyclic tensile strain (CTS) with a wide range of strain magnitudes, frequencies, and durations can be applied on chondrocytes in monolayer. The experimental setup is validated, exactly controllable, and allows studying the cell response in more details [15–19]. Therefore, it provides new insights about loading and cartilage adaptation [20,21].

Several studies on the effects of CTS on chondrocytes have been published within the last 30 years, but up till now, their results have not yet been carried together. With this present review, we now summarized the previous studies on the effect of CTS on chondrocytes. Our review will give insight to the morphological changes of chondrocytes exposed to CTS, and to its influences on cell viability and proliferation. Our focus was set on changes in extracellular matrix (ECM) gene expression, and protein synthesis in response to CTS. Furthermore, we considered factors that induce catabolic effects, like proteases and pro-inflammatory cytokines, or anabolic effects, like growth factors. We compared different loading protocols with different strain magnitudes, loading frequencies, and loading duration. Also, we tried to differentiate the anabolic and catabolic loading protocols. Besides, several indications exist regarding the effect of CTS on chondrocytes in an inflammatory environment. In conclusion, the purpose of our review was a) to summarize the current knowledge about the effect of CTS on major cartilage ECM proteins and molecules, b) to identify loading protocols that are either anabolic or catabolic, and c) to outline what are the strengths and weaknesses of the two-dimensional *in vitro* cell loading method. This summary would contribute to a better understanding of cartilage adaptation to mechanical loading that is needed to optimize cartilage tissue engineering and rehabilitation process in degenerative joint diseases like osteoarthritis.

## Methods

**Fig 2:**
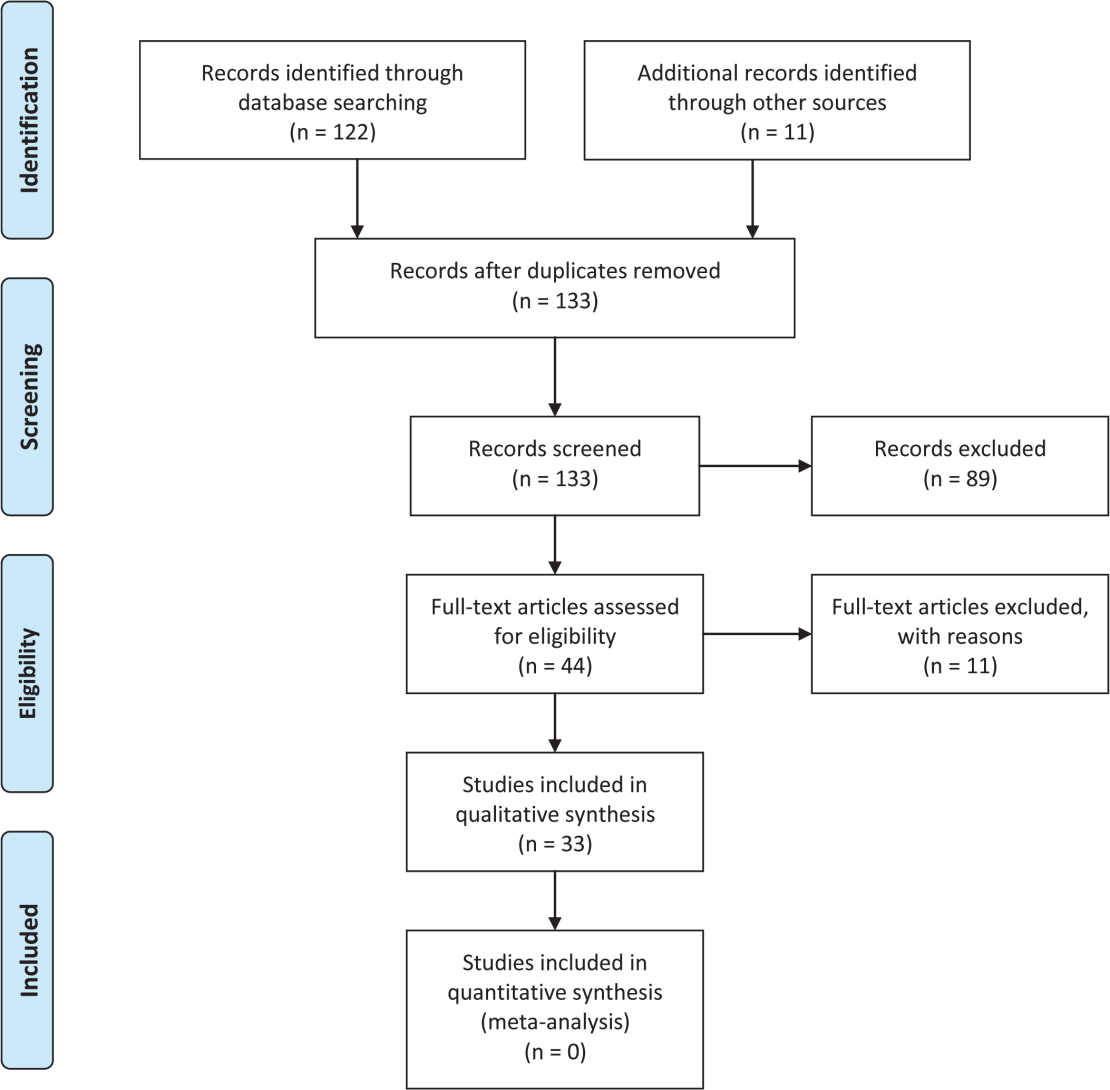
Flowchart of study selection process.

In our systematic literature search in Pubmed, we included the keywords chondrocytes AND cyclic strain OR cyclic tensile strain OR cyclic tensile stretch OR cyclic tensile loading OR intermittent tensile strain OR flexercell OR STREX. “Flexercell” (Flexercell International Corp., Hillsborough, NC, USA) and “STREX” (STREX Inc., Osaka, Japan) are the most used commercially available cell stretching instruments and were therefore included as keywords. This resulted in a total of 122 articles published between 1984 and 2013. Search with google scholar gave 11 additional publications that were not found in Pubmed. These 133 publications were screened for eligibility. Inclusion criteria were 1) cells must be chondrocytes from healthy hyaline cartilage and 2) loading characteristic must be CTS in monolayer culture (Fig. 2, S1 Checklist).

## Results

From the 133 publications, 89 were excluded because three were review articles, and the others (n = 86) used different cell types (e. g. fibrochondrocytes, fibroblasts, annulus fibrosus cells, meniscal cells, chondrocytic cell lines, chondrosarcoma cells) and/or different loading types (compression, three-dimensional loading, shear), or finite element analysis. After careful screening of the remaining 44 scientific papers, eight publications were excluded because there was insufficient information about the loading protocol. Two others were excluded because the chondrocytes were not from healthy joints; and one was also excluded because there was a discrepancy between the data described in the text and the same data presented in a figure. In the total 33 publications reviewed (Table 1), chondrocytes from animal or human hyaline joint, rib cage or endplate cartilage were investigated in all of them. Cells were cultured in monolayer and exposed to CTS. The publications cover a wide range of loading protocols. As response to these, intra- and extracellular effects were examined.

**Table 1:** Included studies.

Author	Cell type	Culture plate coating	Strain magnitude	Loading duration	Loading frequency	Investigated parameters
Agarwal et al. 2004 [76]	14–16 months old rabbits; chondrocytes from shoulder and knee joint articular cartilage	Pronectin	4, 8, 12, 15, 18%	15, 30, 60, 90 min, 2, 4, 8, 12, 16, 18, 24 h	0.05 Hz	iNOS, NO, NF-kB
Akagi et al. 2006 [30]	10 months old calves; chondrocytes from metacarpophalangeal joint articular cartilage	Not specified	5%	6 h	0.17 Hz	Cell viability, Proteoglycan, oxLDL
Doi et al. 2008 [38]	7 days old Wistar Rats; chondrocytes from femoral condyle articular cartilage	Collagen I	7%	3, 6, 12, 24, 36 h	0.5 Hz	Morphology, collagen II mRNA, aggrecan mRNA, cathepsin B, MMP-13, IL-1β
Dossumbekova et al. 2007 [20]	10–12 weeks old Sprague-Dawley rats; chondrocytes from knee joint articular cartilage	Collagen I	3%	10, 30, 60, 90min	0.05 Hz	NF-kB
Fukuda et al. 1997 [31]	10 months old calves; chondrocytes from metacarpophalangeal joint articular cartilage	Collagen I	5, 15%	3 min, 1, 18, 24 h	0.17 Hz, every 6 min stretch for 3 s	Cell number, DNA synthesis, morphology, proteoglycan
Gassner et al. 1999 [52]	6–7 pounds, young adult New Zealand white rabbits; chondrocytes from shoulder and knee joint articular cartilage	Pronectin	20%	2, 4, 12, 24, 48, 72, 96 h	0.05 Hz	Proteoglycan, iNOS, NO
Gassner et al. 2000a [77]	3–4 kilograms New Zealand white rabbits; chondrocytes from shoulder and knee joint articularcartilage	Pronectin	20%	2, 4, 12, 24, 48, 72, 96 h	0.05 Hz	iNOS, NO
Gassner et al. 2000b [48]	5–6 pounds, young adult New Zealand white rabbits; chondrocytes from shoulder and knee joint articular cartilage	Pronectin	5%	24 h	0.05 Hz	Proteoglycan, TGF-β1, NO
Holmvall et al. 1995 [14]	4–6 months old calves; chondrocytes from metacarpophalangeal joint articular cartilage	Amino, Collagen II	24%	1, 3, 20 h	0.25 Hz	Collagen II mRNA, aggrecan mRNA
Honda et al. 2000 [34]	4 weeks old Japanese white rabbits; chondrocytes from the surface and middle zones of knee articular cartilage	Collagen II	23%	12 h	0.5 Hz	Morphology, proteoglycan, collagen II (Immunhistochemistry), MMP-1, MMP-3, MMP-9, IL-1β, TNF-α
Huang et al. 2007 [37]	1 weeks old pigs; chondrocytes from patellofemoral groove and femoral condyle articular cartilage	Collagen I	10%	1, 3, 6, 12, 24 h	0.5 Hz	Morphology, aggrecan mRNA, collagen II mRNA, TGF-β1, MMP-1, COX-2, PGE_2_, NO
Iimoto et al. 2005 [36]	7 days old Wistar rats; chondrocytes from knee joint articular cartilage	Collagen I	7%	36 h	0.5 Hz,	Morphology, collagen II mRNA, aggrecan mRNA, COX-2
Iscru et al. 2008 [21]	10–12 weeks old Sprague-Dawley rats; chondrocytes from knee joint articular cartilage	Collagen I	3%	24 h	0.25 Hz	Morphology, β-actin
Kamiya et al. 2009 [57]	6–9 months old (100–110 kilograms) female pigs; chondrocytes from temporomandibular joint condylar articular cartilage	Laminin	7, 21%	12, 24, 48 h	0.5 Hz	Superficial zone protein, TGF-β1, Il-1β
Kawakita et al. 2012 [22]	Human knee primary OA chondrocytes from femoral condyle articular cartilage and non-arthritic femoral neck articular cartilage	Collagen I	2, 5, 10%	12 h	0.25 Hz	Collagen II mRNA, aggrecan mRNA, proteoglycans
Long et al. 2001 [53]	10–12 pounds, young adult New Zealand white rabbits; chondrocytes from shoulder and knee joint articular cartilage	Pronectin	6%	4, 8, 24, 48, 72, 96 h	0.05 Hz	Proteoglycan, MMP-1, TIMP-1, TIMP-2, iNOS, COX-2, NO, PGE_2_
Madhavan et al. 2006 [29]	14–16 weeks old Sprague-Dawley rats; chondrocytes from shoulder and knee joint articular cartilage	Collagen I	3%	4, 8, 12, 16, 24, 36, 48 h	0.25 Hz	Viability, proliferation, aggrecan mRNA, MMP-9, MMP-13, iNOS, COX-2, NO
Marques et al. 2008 [60]	Newborn rats; chondrocytes from mandibular condyles cartilage	Collagen I	7%	4 h	0.33 Hz	Fibronectin, IGF-1, IGF-2
Matsukawa et al. 2004 [47]	10 months old calves; chondrocytes from metacarpophalangeal joint condyle articular cartilage	Collagen I/II, Fibronectin, Albumin	7%	24 h	0.167 Hz	Proteoglycan, iNOS, NO
Ohno et al. 2005 [24]	4 weeks old Wistar strain rats; chondrocytes from rib growth plate cartilage	Collagen II	12%	6, 12, 24 h, some days	0.5 Hz	Collagen II mRNA, collagen X mRNA, aggrecan mRNA, TGF-β1
Perera et el. 2010 [32]	12–14 weeks old female Sprague-Dawley rats; chondrocytes from knee joint articular cartilage	Collagen I	6%	90 min a day, 2 days	0.25 Hz	Proliferation, cMyc, SOX-9, VEGF, ERK1/2, MEK ½, IL-1β
Ru-Song et al. 2012 [13]	2 weeks old rats; chondrocytes from mandibular condyle articular cartilage	Not specified	10%	0, 1, 6, 12, 24 h	0.167 Hz	Morphology, collagen II mRNA, aggrecan mRNA
Shimizu et al. 2004 [33]	7 days old Wistar Rats; chondrocytes from knee joint articular cartilage	Collagen I	7%	12, 16, 24 h	0.5 Hz	Morphology, collagen II mRNA, aggrecan mRNA, fibronectin
Tanaka et al. 2005 [25]	4 weeks old male Wistar strain rats; chondrocytes from rib cage growth plate cartilage	Collagen II	7, 12%	12 h, 24 h	0.5 Hz	DNA Synthesis, morphology, collagen II, proteoglycan,
Tanimoto et al. 2009 [75]	4 weeks old Japanese white rabbits; chondrocytes from knee joint articular surface	Not specified	22.80%	6, 12, 24, 48 h	0.5 Hz	HYAL1, HYAL2, IL1-β, TNF-α
Tanimoto et al. 2011 [45]	6–9 months old (100–110 pounds) pigs; chondrocytes from mandibular condyle articular cartilage	Not specified	7, 21%	0, 6, 12, 24, 48 h	0.5 Hz	Superficial zone protein
Thomas et al. 2011 [46]	7 days old calves; chondrocytes from metacarpophalangeal joint articular cartilage	Pronectin	7.5%	30 min	1 Hz	Collagen II mRNA, aggrecan mRNA, MMP-3, MMP-13, ADAMTS-4, ADAMTS-5, SOX-9, c-fos, c-jun, Lef-1, Wnt3A
Ueki et al. 2008 [23]	4 weeks old Wistar rats; chondrocytes from rib growth plate cartilage	Collagen II	3%	12 h	0.03, 0.5, 2.5 Hz	DNA Synthesis, proteoglycan, collagen II
Wang et al. 2011 [26]	2–3 years old steers; chondrocytes from knee articular cartilage	Fibronectin	16%	12, 24, 48 h	0.5 Hz	Morphology, cell viability, collagen II mRNA, aggrecan mRNA, gene expression in 23 different genes
Xu et al. 2000 [27]	6–7 pounds, young adult New Zealand white rabbits; chondrocytes from shoulder and knee articular cartilage	Pronectin	6%	4, 24, 48, 72, 96 h	0.05 Hz	Cell viability, collagen II mRNA, aggrecan mRNA, proteoglycans, versican, biglycan, MMP-1, TIMP-1, TIMP-2, iNOS, COX-2, NO, PGE_2_
Xu et al. 2011 a [97]	160–180 grams Sprague-Dawley rats; chondrocytes from lumbar spine end-plate articular cartilage	Collagen I	10%	3, 6, 12, 24, 36, 48 h	0.5, 1, 1.5, 2 Hz	Morphology, TGF-β1
Yamazaki et al. 2003 [74]	10 months old calves; chondrocytes from metacarpophalangeal joint condyle articular cartilage	Not specified	17%	24 h	3 s strain—6 min relaxation, 0.5 Hz	Hyaluronan
Yorimitsu et al. 2008 [28]	7 days old Wistar rats; chondrocytes from epiphyseal femoral condyle articular cartilage	Collagen I	7%	24, 48 h	0.5 Hz	Cell viability, iNOS, NO

Listing of included studies with description of cell source, culture plate coating, loading protocol and parameters that were investigated.

### Cells

In almost all cases, hyaline articular chondrocytes from healthy animal joints were investigated (shoulder (n = 7), knee (n = 22), and temporomandibular joints (n = 4) of rabbits, rats, pigs, and a steer; metacarpophalangeal joints (n = 6) of calves; and spine endplate cartilage (n = 1) of rats). In one case, healthy human articular chondrocytes from the femoral head were investigated [22]. These samples were obtained from patients undergoing femoral head replacement surgery after neck fracture. In three cases, chondrocytes from the rib-cages of rats were used [23–25].

### Cell Culture

Cells were isolated by enzymatic digestion and seeded in monolayer on culture plates with deformable membranes. Cells were cultured to 80–100% confluence. Membranes were either coated with collagen I (n = 14), collagen II (n = 6), pronectin (n = 7), fibronectin (n = 2), laminin (n = 1), or albumin (n = 1). In five cases, coating was not specified. Ten publications investigated the effects of CTS on chondrocytes in an inflammatory environment. Here, interleukin-1β (IL-1β) or tumor necrosis factor α (TNF-α) were added to the culture media. Primary chondrocytes until 3^rd^ passage were used in all the studies. In passaged cells, the expression of collagen II was monitored to ensure that the chondrocytes maintained their phenotype. The cell isolation procedure, the number of cells seeded and the time of culture until the loading protocol started, varied between the studies. One has to consider that these factors might influence the starting situation of the cells, and therefore, influence their response to the loading intervention even though the loading protocol was identical.

### Loading Device

The commercially available Flexercell strain unit (types FX2000, FX3000, FX4000 or FX5000; n = 32) or a custom made strain instrument (n = 1) [26] were used as loading devices. All loading devices were vacuum driven. The vacuum pulled the deformable membranes of the culture plates over loading posts (Fig. 1B). Once the vacuum was released, the membranes returned to their original shape. The cells that were attached on the membranes followed the deformation of the membrane and thereby experienced tensile strain. Depending on the setup, biaxial and uniaxial forms of strain can be applied.

### Loading Protocols

Loading protocols encompassed strains of the membranes of 0–23% at frequencies of 0.03–2.5 Hz and loading durations of 0–96 h. The most commonly used loading protocol was 7% strain at 0.5 Hz for 24 h, which was investigated in 6 studies. Strain characteristics were biaxial in 11 publications. In 22 cases, it remained unclear if uni- or biaxial strain characteristics were used. In almost all cases (n = 29) sinusoidal waveforms were applied on the cells. Four studies did not specify the waveform. Other loading characteristics, like the loading rate or different-shaped loading curves (e. g. triangle wave, square wave) haven´t been investigated yet. Unless otherwise indicated, differences in cell response were always represented as differences between loaded and unloaded control cells. Control cells were cultured either on rigid-bottomed (n = 11) or on flexible-bottomed (n = 21) culture plates. In total, control cells and loaded cells were cultured for the same period of time, however, control cells remained unloaded (n = 32; not specified: n = 1).

### Data Collection

With the following exceptions, all authors collected the data immediately after the last loading cycle. Madhavan et al. (2006) investigated different intervals of loading and resting (CTS/rest for 4/20, 8/16, 12/12, 16/8, 29/4, 24/0 h), while Thomas et al. (2011) analyzed the parameters immediately after the last loading cycle and after a 4 h recovery period.

### Cell viability and Proliferation

#### Viability

Viability was assessed in six studies; in five of these studies, loading did not affect cell viability. In details, more than 90% of cells remained alive after 48 h of continuous CTS with 16% strain at 0.5 Hz [26]. Accordingly, Fukuda et al. (1997) reported that the cell number after two very differing loading protocols (24 h, 5%, 0.003 Hz, and 35 h, 17%, 0.17 Hz) did not vary from unloaded samples. Furthermore, no significant cell death was observed after 24 h of 5% CTS at 0.05 Hz [27] and after 24 and 48 h of 7% CTS at 0.5 Hz [28]. During 24 h of experimentation with 3% CTS at 0.25 Hz, no differences in cell viability to unloaded cells occurred [29]. However, Akagi et al. (2006) were the only group showing changes in cell viability due to loading. Here, 24 h of 5% strain at 0.17 Hz decreased cell viability significantly to 84% [30].

#### Proliferation/DNA Synthesis

Cell proliferation was assessed in five studies by measuring the short-term change in metabolism (DNA synthesis) with the incorporation of [^3^H-thymidine] into the cells [25,31], by a MTT cell proliferation assay [32], and by a Pico-Green dsDNA quantitation kit [23] (Table 2). In one case, the authors described that the proliferation rate did not differ after loading, but the method, used to assess the proliferating rate, was not specified [29]. Chondrocytes under CTS with a frequency of 0.003 or 0.03 Hz did not alter their DNA synthesis compared to unloaded samples [23,31]. However, higher frequencies (0.17–2.5 Hz) increased cell proliferation [23,25,31,32]. These observations suggest that loading frequency has a greater influence on cell proliferation than other loading parameters, like for example strain magnitude. Cell viability remained mainly unaffected by CTS. However, more studies investigating viability and proliferation are needed to make more precise statements.

**Table 2:** Effects of CTS on cell proliferation.

Frequency	Loading duration	Strain magnitude	DNA Synthesis	Reference
0.003 Hz	24 h	5%	═	[31]
0.03 Hz	12 h	3%	═	[23]
0.17 Hz	24 h	17%	↑	[31]
0.25 Hz	90 min, 2 days	6%	↑^a^	[32]
0.5 Hz	12 h	3%	↑	[23]
	24 h	7%	↑	[25]
	24 h	12%	↑	[25]
2.5 Hz	12 h	3%	↑	[23]

Effects of CTS on cell proliferation (DNA synthesis) relative to unloaded controls, sorted by loading frequency

═ Levels of loaded cells were unchanged relative to unloaded cells

↑ Levels of loaded cells were increased relative to unloaded cells

^a^ In this study cell proliferation was examined by a MTT assay, whereas the others measured the short term change in metabolism (DNA synthesis) with the incorporation of [3H-thymidine] into the cells.

### Cell morphology

The morphology of chondrocytes with and without CTS was described in 12 publications. Chondrocytes attached 2–3 days after plating on deformable membranes and exhibited a polygonal and spread shape [13,26,33,34]. At confluence, cell layer displayed the characteristic “cobblestone” appearance [13,31,35,36]. Cells adhered well on the culture plate surface and were randomly orientated and distributed [35,37]. Several authors reported that after loading with CTS, cells exhibited a more elongated cell shape and aligned perpendicular to the loading direction [34–36]. However, others observed no distinct orientation [13,25,31,38]. A possible explanation for this might be the particular strain characteristic. Huang et al. (2007) described no differences in the alignment of the cells at the area above the loading post, where there is equibiaxial strain. However, they found an alignment perpendicular to the load direction at the edge of the wells, where the membranes are gliding over the rim of the loading posts with uniaxial strain [37]. The reason for this alignment away from the loading direction might among other things be related to cytoskeletal remodeling. From a mechanical point of view, the adjustment of the cytoskeleton and of the cell shape may represent a strategy to better withstand the loading. This may be a stretch avoidance reaction and might protect cells from longitudinal loading [39,40]. Interestingly, one of the reviewed publications reported that the actin cytoskeleton was remodeled in response to CTS [21]. Unfortunately, in most of the selected publications, it was not specified if strains were equibiaxial or uniaxial or which position on the wells the investigated cells were from to better address this point.

In addition to gross cell morphology and cytoskeletal arrangement, one publication investigated the effects of CTS on the surface topography at the ultra-structural level. They reported that native chondrocytes show a relatively smooth cell surface [21]. However, under 3% CTS at 0.25 Hz for 24 h, average particle density and average granular size increased [21]. The authors interpreted the results as an increased secretion of pericellular components and an up-regulation of cell-surface receptors for mechanotransduction due to the loading stimulus. Changes in cell morphology, especially during monolayer culture, are associated with phenotypic alterations of chondrocytes [41] and different pattern of protein synthesis [42]. If and how CTS influences these phenotypic changes is currently not known. However, Wang et al. (2011) showed that CTS of 16% for 48 h had a positive influence on hyperthrophic markers, like collagen X [26] and suggested that high CTS induced an arthritic phenotype. On the other side, Ohno et al. (2005) proposed that CTS of 12% for a maximum of 24 h reduced hypertrophic differentiation [24]. Thus, further investigation is needed to reveal which loading protocols rather induce phenotypic changes and which protocols preserve the phenotypic homeostasis.

### Extracellular Matrix

#### Collagen II and Proteoglycans

The extracellular matrix proteins collagen II and proteoglycans were investigated most frequently in response to CTS, corresponding to their prominent appearance among all proteins in articular cartilage. Several of the selected studies deal with the effect of CTS on collagen II (n = 9) and aggrecan (n = 11) at the mRNA level. The total collagen synthesis was measured by [2,3-³H]proline incorporation and was investigated in two cases [23,25]. One study analyzed collagen II at the protein level using immunostaining of chondrocytes after CTS [34]. None of the other collagens prominent in cartilage (e. g. collagen III, V, VI, IX, X, XI, XII, and XIV) [43,44] have yet been investigated in response to CTS. In three articles, the cartilage-unspecific gene collagen I [45] or the hypertrophy-associated gene collagen X [24,26] were analyzed, but only to control the phenotype and dedifferentiation state of the chondrocytes. The total proteoglycan synthesis was measured as incorporation of [^35^S]-labeled proteoglycans into the cells and the medium (n = 10). One study analyzed biglycan and versican [27]. However, no effects of CTS on the mRNA levels of these two proteins were observed. Other GAGs or proteoglycans, like decorin, fibromodulin and lumican were not yet investigated in response to CTS. Interestingly, the response pattern of collagen II and proteoglycans to CTS were similar and therefore were discussed simultaneously in the following. Furthermore, the results of collagen II and proteoglycans are sorted according to the loading duration, since this parameter seemed to influence the cellular response to CTS substantially.

#### Collagen II and Proteoglycans—RNA Level

Compared to unloaded cells [37,46] and to mRNA levels before loading [13], collagen II and aggrecan were not altered after loading regimes that were shorter than 60 min (Table 3). However, Thomas et al. (2011) made an interesting observation: Immediately after a 30 min loading, mRNA levels were not changed compared to those of unloaded cells [46]. However, after a recovery of 4 h after the loading session, mRNA levels were elevated significantly [46], indicating a certain delay due to the process of transcription. Also, one can assume that loading regimes under 60 min might stimulate cells to express collagen II or aggrecan mRNA, but it can only be measured at later time points.

The reviewed publications further showed that collagen II and aggrecan mRNA levels were increased when loading lasted between 3 or 6 h [13,14,37] (Table 3). Hence, when loading exceeds 3 h, the elevated levels can be measured immediately after the loading. Most likely, the detected transcripts then result from early loading. The response at loading durations of 3 and 6 h, however, was strain magnitude dependent: strain magnitudes of 7% did not change mRNA levels [33,36,38] while higher strain magnitudes (10 and 24%) elevated collagen II and aggrecan mRNA levels [13,14,37].

**Table 3:** Effects of STC on collagen II and aggrecan mRNA.

Loading duration	Strain magnitude	Frequency	Collagen II mRNA level	Aggrecan mRNA level	Reference
0.5 h	7.5%	1 Hz	═	═	[46]
	7.5%	1 Hz	↑^a^	↑^a^	[46]
1 h	10%	0.5 Hz	═	═	[37]
	10%	0.17 Hz	═^b^	═^b^	[13]
3 h	7%	0.5 Hz	═	═	[38]
	10%	0.5 Hz	↑	↑	[37]
	24%	0.25 Hz	↑	↑	[14]
6 h	7%	0.5 Hz	═	═	[33]
	7%	0.5 Hz	═	═	[38]
	10%	0.17 Hz	↑^b^	↑^b^	[13]
12 h	7%	0.5 Hz	↑	═	[33]
	7%	0.5 Hz	═	═	[38]
	7%	0.5 Hz	═	═	[36]
	10%	0.5 Hz	↓	═	[37]
	10%	0.17 Hz	↓^b^	═^b^	[13]
	16%	0.5 Hz	═	═	[26]
16 h	7%	0.5 Hz	↓	↓	[33]
18 h	7%	0.5 Hz	↓	↓	[36]
24 h	6%	0 05 Hz	═	═	[27]
	7%	0.5 Hz	↓	↓	[33]
	7%	0.5 Hz	═	═	[38]
	7%	0.5 Hz	↓	↓	[36]
	10%	0.5 Hz	↓	═	[37]
	10%	0.17 Hz	↓^b^	↓^b^	[13]
	16%	0.5 Hz	═	═	[26]
36 h	7%	0.5 Hz	═	═	[38]
48 h	6%	0.05 Hz	═	═	[27]
	16%	0.5 Hz	↓	═	[26]
72 h	6%	0.05 Hz	═		[27]

Effects of CTS on collagen II and aggrecan mRNA level in articular chondrocytes, sorted by loading duration

↓ mRNA levels of loaded cells were decreased relative to unloaded cells

═ mRNA levels of loaded cells were unchanged relative to unloaded cells

↑ mRNA levels of loaded cells were increased relative to unloaded cells

^a^ mRNA levels measured after a 4 h recovery instead of immediately after the loading

^b^ mRNA levels after loading were compared to levels before loading

**Fig 3:**
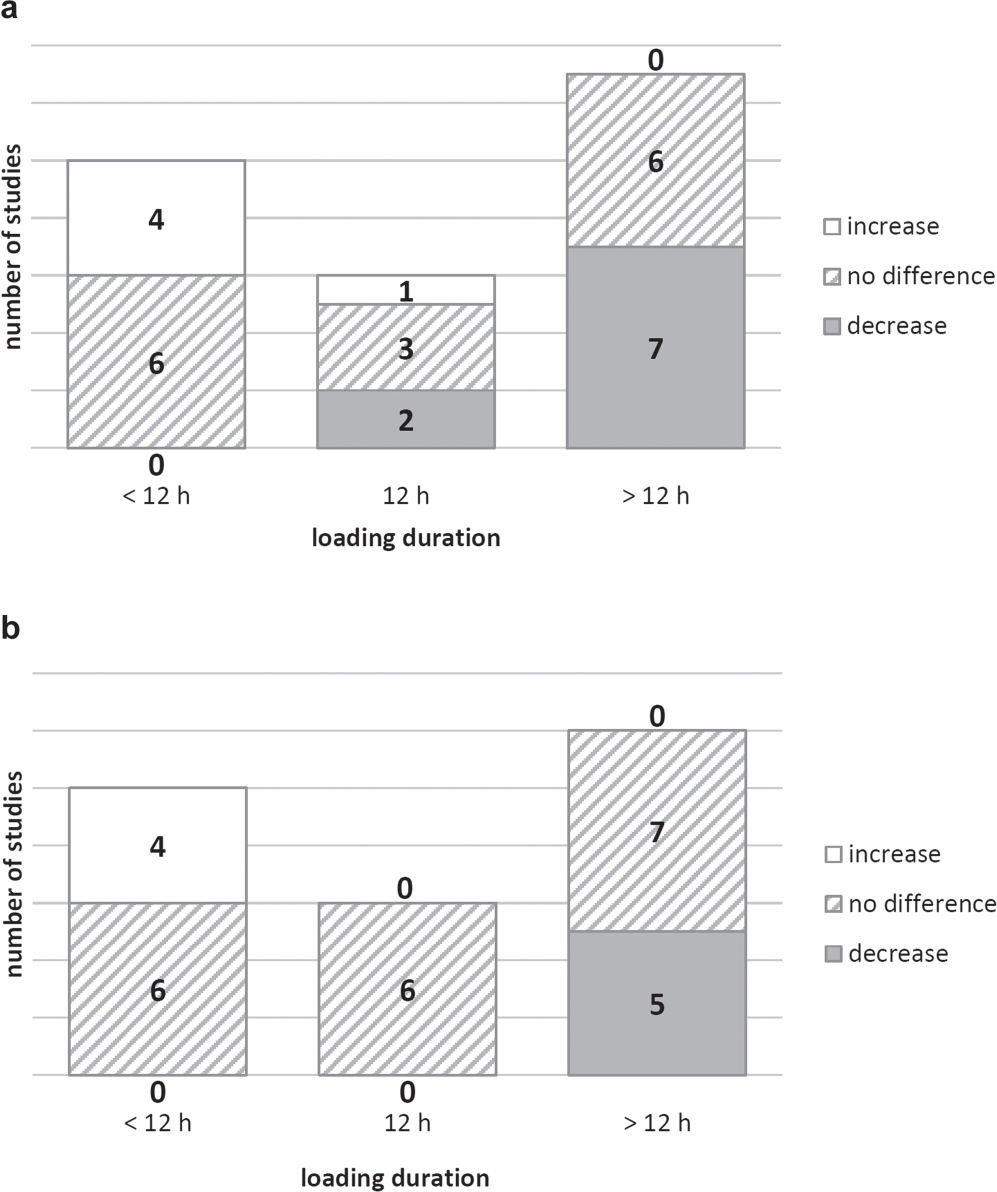
Effects of CTS on collagen II and aggrecan mRNA levels. Number of experiments investigating the effects of CTS on collagen II (**a**) and aggrecan (**b**) mRNA levels and summary of the effects on these parameters. Results were divided by loading duration (less than 12 h of loading, exactly 12 hours of loading, longer than 12 hours of loading) and studies were separated into: studies that found an increase in mRNA, studies that found no difference relative to control levels and studies that found a decrease of mRNA relative to control level.

At a loading duration of 12 h, differing responses in collagen II mRNA were observed [13,26,33,36–38] (Table 3, Fig. 3). Aggrecan mRNA levels of cells loaded for 12 h were not altered compared to the levels of unloaded chondrocytes [13,26,33,36–38]. Thereafter (16–72 h of loading), cell response reversed and mRNA levels of both proteins were mostly down-regulated [13,26,33,36,37] (Table 3, Fig. 3).

#### Collagen II and Proteoglycans—Protein Level

Total collagen synthesis was only measured in two studies and both showed an increase in response to 12 and 24 h CTS [23,25] (Table 4).Honda et al. (2000), however, observed a decreased staining intensity for collagen II in immunostained chondrocytes after high magnitude tensile strain (23%) for 12 h. The decrease was particularly obvious in the middle of the wells, the area which was subjected to the greatest load [34]. More information is available about the proteoglycan synthesis in response to CTS. One study showed that high magnitude tensile strain (23%) at 0.5 Hz decreased total proteoglycan synthesis after 3 and 6 h of loading, but the differences in unloaded controls was abrogated after 12 h loading [34]. This documents a possible desensitization of the cells to the altered mechanical environment. Ueki et al. (2008) demonstrated that low frequency (0.03 Hz) did not affect the proteoglycan synthesis, whereas higher frequencies (0.5 and 2.5 Hz) increased the synthesis of proteoglycans. After mechanical loading with a duration of 24 h, diverging results were obtained which may not only be attributed to different loading protocols, but also to different coatings of the culture plates (Table 4). In particular, Matsukawa et al. (2004) observed that on fibronectin coated plates, proteoglycans increased. Whereas on collagen I coated culture plates, proteoglycans were decreased [31,47]. On pronectin coated plates, no changes between unloaded cells and cells under CTS were observed [48]. The differences might be explained by the integrin-mediated attachment of cells to the coated protein [49]. Integrins transmit signals between the cell and the ECM during mechanical loading [50]. It is known that chondrocytes express different integrins in response to different coatings [51]. Therefore, on different coatings, the integrin-mediated effects might change cell behavior and protein synthesis in response to CTS. However, when loading lasted longer than 24 h, proteoglycan synthesis was reduced regardless of protein coating of strain magnitude [27,52,53] (Table 4).

**Table 4:** Effects of CTS on Proteoglycan synthesis.

Loading duration	Strain magnitude	Frequency	Culture plate coating	Collagen synthesis	Proteoglycan synthesis	Reference
6 h	5%	0.17 Hz	Fibronectin		═	[30]
	23%	0.5 Hz	Collagen II	↓^a^	↓	[34]
12 h	3%	0.03 Hz	Collagen II	═	═	[23]
	3%	0.5 Hz	Collagen II	↑	↑	[23]
	3%	2.5 Hz	Collagen II	↑	↑	[23]
	5%	0.17 Hz	Fibronectin		↓	[30]
24 h	5%	0.17 Hz	Fibronectin		↓	[30]
	5%	0.003 Hz	Collagen I		↑	[31]
	5%	0.05 Hz	Pronectin		═	[48]
	6%	0.05 Hz	Collagen I		↓	[27]
	7%	0.17 Hz	Collagen I		↓	[47]
	7%	0.17 Hz	Fibronectin		↑	[47]
	7%	0.5 Hz	Collagen II	↑	↑	[25]
	12%	0.5 Hz	Collagen II	↑	↑	[25]
	17%	0.17 Hz	Collagen I		↓	[31]
48 h	6%	0.05 Hz	Collagen I		↓	[27]
	20%	0.05 Hz	Pronectin		↓	[52]
72 h	6%	0.05 Hz	Collagen I		↓	[27]
	6%	0.05 Hz	Pronectin		↓	[53]
	20%	0.05 Hz	Pronectin		↓	[52]
96 h	6%	0.05 Hz	Pronectin		↓	[53]

Effects of CTS on total collagen and proteoglycans synthesis, sorted by loading duration

↓ Proteoglycan or collagen synthesis of loaded cells was decreased relative to unloaded cells

═ Proteoglycan or collagen synthesis of loaded cells was unchanged relative to unloaded cells

↑ Proteoglycan or collagen synthesis of loaded cells was increased relative to unloaded cells

^a^ Assessed as intensitiy of immunostaining

Several studies have shown that the exposure of cells to an inflammatory stimulus (IL-1 or TNF-α) strongly down-regulated proteoglycan levels [27,48,52,53]. This reduction might occur via an inhibition of aggrecan mRNA [29] or an IL-1 and TNF-α-induced activation of proteases like ADAMTSs or MMPs, which in turn cleave proteoglycans [54]. It is noticeable that low CTS (3 or 5% respectively) at several durations counteracted the aggrecan mRNA inhibition [29] and partly restored the synthesis of proteoglycans [48]. This proposes a beneficial effect of certain loading protocols on the cartilage ECM in already inflamed joints.

Summing up, the loading duration might be the main stimulus for collagen II and aggrecan mRNA expression. Thereby, within a window of 12 h of loading, chondrocytes increase the mRNA expression, mainly independently of strain magnitude and loading frequency. Thereafter, cells possibly gradually adapt to the altered mechanical environment and down-regulate collagen II and aggrecan expression. Considering the time delay between gene transcription and protein synthesis, the elevated levels of mRNA within 12 h is reflected at the protein level at later time points: For example, 7% strain and 0.5 Hz increased collagen II mRNA after 12 h [33] and the collagen synthesis after 24 h [25]. And the decrease in aggrecan mRNA when loading lasts longer than 16 h, is in accordance with a reduced proteoglycan synthesis after 48 h of loading. However, it has been shown that changes in the biosynthesis may not be related solely to changes in mRNA expression [55]. While aggrecan and collagen II mRNA were up-regulated during the initial 0.5 h of static compression and decreased during the following 4–24 h, the synthesis of aggrecan and collagen protein decreased more rapidly already after 0.5 h [55]. However, none of the reviewed studies investigated collagen II or proteoglycans at both the mRNA and the protein level. Furthermore, the mRNA level alone does not give information about how the extracellular matrix is adapted in response to the loading. The secretion and assembly of protein into the extracellular space is essential to change the mechanical properties of the tissue. Therefore, when investigating extracellular matrix proteins, like collagen II or proteoglycans, further investigations should include not only mRNA analysis but especially a detailed analysis of the extracellular amount and spatial distribution pattern of the proteins. Hence, it would be of interest to distinguish between soluble protein that is released into the supernatant and protein that is embedded into extracellular structures.

#### Superficial Zone Protein

The superficial zone protein contributes to the lubrication function of the surface layer or articular cartilage which is essential for nearly frictionless gliding of the articulating joint partners under motion [56]. CTS of 7% upregulated mRNA levels of superficial zone protein after 12, 24 and 48 h compared to levels before loading [45] and compared to unloaded cells [57]. Higher strains (21%) elevated mRNA levels after 12 h loading compared to levels before loading [45] and compared to unloaded cells [57]. Nevertheless, it decreased under control levels after 48 h of loading. Accordingly, immunoblot analysis revealed that superficial zone protein levels increased under 7% strain and decreased under 21% strain [57]. The results suggest that moderate loading supports lubrication and low-friction-motion by increasing expression of superficial zone protein in chondrocytes. Mechanical overloading, however, inhibits the expression and synthesis and thereby provokes cartilage degradation under motion since lubrication function is disturbed.

#### Fibronectin

Fibronectin connects collagen fibers and other ECM proteins [58]. It is linked to the cell membrane through integrins and might transmit forces from the ECM to the chondrocyte [59]. CTS at 7%, 0.33 Hz and 0.5 Hz, for 4, 12 and 24 h increased the fibronectin mRNA levels in comparison to non-loaded chondrocytes [33,60]. This suggests that tissue adaptation in response to moderate CTS also comprises the production of molecules that are involved in matrix-cell connection and mechanical signal transmission, like fibronectin. To our knowledge, other non-collagenous matrix proteins have not yet been investigated in response to CTS in monolayer. However, it has been shown in three-dimensional agarose constructs that for example the cartilage oligomeric matrix protein (COMP) was increased in response to cyclic tension in chondrocytes [61]. Further investigation is needed to understand the complex interplay of mechanical signals and matrix adaptation. Information about the effect of two-dimensional CTS on non-collagenous proteins like the adhesive glycoproteins thrombospondin or chondroadherin, as well as proteins that connect the cartilage network like COMP, proline-rich-protein, leucine-rich-protein or matrilins (matrilin-1, -2, -3, -4) are missing. Moreover, the interaction of ECM proteins under tensile strain and the influence of different loading protocols on chondrocytes in an inflammatory environment remain to be investigated.

### Extracellular Matrix Supporting and Degrading Factors

#### Factors that Promote Matrix Synthesis

Insulin like growth factors (IGFs) and the transforming growth factor β (TGF-β) promote anabolic activities in chondrocytes and stimulate the gene expression of collagen II and aggrecan [62–64]. Furthermore, these factors regulate chondrocyte proliferation and differentiation [65]. Marques et al. (2008) showed that 7% CTS for 4 h at 0.33 Hz elevated the expression of IGF-1 and IGF-2. The mRNA expression of TGF-β1 was increased by several loading protocols ranging from strains of 5–12%, from 12–48 h and at frequencies of 0.05 and 0.5 Hz [24,37,48,57] (Table 5). The increased expression of IGF and TGF-β1 due to CTS might in turn support the synthesis of collagen II and aggrecan after these loading protocols.

**Table 5:** Effects of CTS on TGF-β1.

Loading duration	Strain magnitude	Frequency	TGF-β1	Reference
3 h	10%	0.5 Hz	═	[37]
6 h	12%	0.5 Hz	═	[24]
12 h	7%	0.5 Hz	↑	[57]
	10%	0.5 Hz	═	[37]
	12%	0.5 Hz	↑	[24,57]
24 h	5%	0.05 Hz	↑^a^	[48]
	7%	0.5 Hz	↑	[57]
	10%	0.5 Hz	↑	[37]
	12%	0.5 Hz	═	[57]
	12%	0.5 Hz	↑	[24]
48 h	7%	0.5 Hz	↑	[57]
	12%	0.5 Hz	═	[57]

Effects of CTS on TGF-β1 relative to unloaded controls, sorted by loading duration

═ mRNA levels of loaded cells were unchanged relative to unloaded cells

↑ mRNA levels of loaded cells were increased relative to unloaded cells

^a^ TGF-β activity determined with a bioassay

#### Degradation and Loss of Matrix Macromolecules

The degradation of the ECM of cartilage is accomplished by proteases. Collagenases, like metalloproteinase-1 (MMP-1), MMP-3, MMP-9, and MMP-13 are able to cleave the collagen network [66], whereas aggrecanases, like ADAMTS-4 (a disintegrin and metalloproteinase with a thrombospondin motif 4), and ADAMTS-5 degrade the proteoglycan aggrecan [67]. The hyaluronidases HYAL1 and HYAL2 can cleave hyaluronan, which in turn will destabilize the supramolecular structures and weaken the cartilage [68,69]. These degrading enzymes are regulated inter alia by inflammatory mediators, like IL-1β and TNF-α, which are closely related to matrix breakdown and which also play a significant role in degenerative disease, like osteoarthritis [70–73].

#### Proteases

Low frequencies of 0.05 Hz did not induce catabolic reactions through proteases in chondrocytes [27,53]. However, several studies showed that both the aggrecan- and collagen-degrading proteases cathepsin B [38], MMP-1, MMP-3, MMP-9 and MMP-13 were up-regulated at various protocols with a frequency of 0.5 Hz, strain magnitudes between 7 and 23%, and loading durations between 3 and 48 h [26,34,37,38,46] (Table 6). Here, MMP-13 was already up-regulated after 3 h of CTS and MMP-1 as recently as after 12 h of CTS. The aggrecanases ADAMTS-4 and ADAMTS-5 were still less sensitive to CTS and only up-regulated in one case [46]. It has been shown that in response to 17% CTS, hyaluronan was slightly depolymerized in the supernatant which was further enhanced by increasing frequencies [74]. A reason for this might be that similar strain magnitudes (22.8%) also up-regulated the mRNA level of the hyaluronidases HYAL1 and HYAL2 after 6 and 12 h of loading respectively, which cleave hyaluronan [75].

**Table 6:** Effects of CTS on proteases.

Frequency	Loading duration	Strain magnitude	MMP-1	MMP-3	MMP-9	MMP-13	ADAMTS-4	ADAMTS-5	Reference
0.05 Hz	4–48 h	6%	═						[27,53]
0.5 Hz	1 h	10%	═						[37]
	3 h	7%				↑			[38]
		10%	═						[37]
	6 h	7%				↑			[38]
	12 h	7%				↑			[38]
		10%	═						[37]
		16%		═		═	═	═	[26]
		23%	↑	↑	↑				[34]
	24 h	7%				↑			[38]
		10%	↑						[37]
		16%		═		↑	═	═	[26]
	36 h	7%				═			[38]
	48 h	16%	↑	═	↑	↑	═	═	[26]
1 Hz	0.5 h	7.5%		═ ↑^a^		═ ↑^a^	═ ↑^a^	═ ═^a^	[46]

Effects of CTS on proteases relative to unloaded controls, sorted by loading frequency

═ mRNA levels of loaded cells were unchanged relative to unloaded cells

↑ mRNA levels of loaded cells were increased relative to unloaded cells

^a^ mRNA levels measured after a 4 h recovery instead of immediately after the loading

#### Pro-inflammatory Factors

Two pivotal pro-inflammatory enzymes in cartilage are the inducible nitric oxide synthase (iNOS) and the cyclooxygenase-2 (COX-2). They induce pro-inflammatory actions through the production of nitric oxide (NO) and prostaglandin E_2_ (PGE_2_). Several studies showed that there was no effect of CTS on the iNOS and COX-2 mRNA expression and on their products NO and PGE_2_ when the loading was applied at a frequency of 0.05 Hz [20,27,48,52,53,76,77] (Table 7). Only one study found an increase in iNOS and NO at 0.05 Hz [76]. However, higher frequencies (0.17 and 0.5 Hz) up-regulated especially COX-2 and with increasing loading duration also iNOS, NO and PGE_2_ [26,28,36,37,47]. Matsukawa et al. (2004) reported that CTS stimulated iNOS mRNA only on fibronectin coated culture plates, but not on collagen coating. Furthermore, NO was up-regulated after CTS when the culture plates were coated with fibronectin, whereas NO production was down-regulated on collagen I coating [47].

**Table 7:** Effects of CTS on pro-inflammatory factors.

Frequency	Loading duration	Strain magnitude	iNOS	NO	COX-2	PGE_2_	Reference
0.05 Hz	10 min - 48 h	3–8%	═	═	═	═	[20,27,48,53,76]
	24 h	12–18%	↑	↑			[76]
	2–96 h	20%	═	═			[52,77]
0.17 Hz	6 h	7%		═^a^ ═^b^			[47]
	12 h	7%		↑^a^ ═^b^			[47]
	24 h	7%		↑ ^a^ ↓^b^			[47]
0.5 Hz	01 h	10%		═	↑	═	[37]
	03 h	10%		═	↑	═	[37]
	06 h	10%		═		═	[37]
	12 h	7%			═		[36]
	12 h	10%		↑	↑	═	[37]
	12 h	16%	═	═	↑	═	[26]
	18 h	7%			═		[36]
	24 h	7%	↑	═			[28]
	24 h	7%			↑		[36]
	24 h	10%		↑	↑	↑	[37]
	24 h	16%	↑	↑	↑	↑	[26]
	36 h	7%		═	═		[36]
	48 h	7%	↑	↑			[28]
	48 h	16%	↑	↑	↑	↑	[26]

Effects of CTS on pro-inflammatory factors relative to unloaded controls, sorted by loading frequency

↓ Levels of loaded cells were decreased relative to unloaded cells

═ Levels of loaded cells were unchanged relative to unloaded cells

↑ Levels of loaded cells were increased relative to unloaded cells

^a^ Cells were seeded on fibronectin

^b^ Cells were seeded on collagen I

The exposure of chondrocytes to the pro-inflammatory cytokines IL1-β and TNF-α up-regulated the matrix degrading proteases MMP-1, MMP-9, MMP-13, the pro-inflammatory enzymes iNOS and COX-2, and their products NO and PGE_2_ [27,29,53,76]. Furthermore, IL-1β suppressed cell proliferation [32]. It is thought that IL1-β and TNF-α play an important role in the development of osteoarthritis [71,78]. Two studies showed that IL-1β was not influenced by CTS of 7% for 12 h [38,57]. However, when loading continued up to 24 h or when the strain magnitude was increased (21–23%) IL-1β and TNF-α were significantly up-regulated [34,38,57,75].

#### Beneficial Effect of CTS in an Already Inflamed Environment

To investigate the beneficial potential of CTS in an inflamed environment, cells were exposed to IL-1β or TNF-α and CTS, simultaneously. Interestingly, CTS at strain magnitudes between 3 and 10% and a frequency of 0.05 Hz led to the suppression of IL-1β and TNF-α induced inflammatory effects already after 60 min [20,76]. Furthermore, 4 and 24 h of loading counteracted the IL-1β and TNF-α induced MMP-1, COX-2, and iNOS expression, the production of NO, and the synthesis of PGE_2_ [27,53,76]. The suppression was evident for strains between 2–10%, whereas the strongest effect was observed at 6% strain [27]. CTS of 12%, 15% and 18% strain, however, had no inhibitory effect on IL-1β induced iNOS expression and NO production [76]. Even more, under these higher strain magnitudes cells produced more NO and elevated the iNOS expression itself. Contrary results were reported by Gassner and colleagues (1999), who found a suppression of IL-1β-induced NO production after 12–96 h of CTS with even higher (20%) strains. The reason for this is unclear. Madhaven and colleagues (2006) explored different durations of low CTS (3%, 0.25 Hz) and found out that the effects of the mechanical loading are persistent. Even after the removal of CTS, the IL-1β induced pro-inflammatory gene transcription were diminished for hours [29].

Furthermore, TNF-α and IL-1β suppress actions that can counteract cartilage destruction, such as the expression of tissue inhibitor of metalloproteinase 2 (TIMP-2) and the expression or synthesis of proteoglycans [27,79,80]. CTS at 6% and 0.05 Hz was able to neutralize this suppression [27,53]. They further reported that TIMP-2 levels, although not suppressed by Il-1β, were hyper-induced by a combination of IL-1ß and CTS [27]. TIMP-1 levels, however, were neither altered by TNF-α nor by IL-1ß or CTS [27,53].

In summary, low magnitude CTS (2–10%) was beneficial to already inflamed joints. These effects were persistent even after the removal of CTS. Interestingly, in a non-inflammatory environment CTS between 12 and 18% mimics the effects of the inflammatory mediator IL-1β and induces similar reactions to those found in osteoarthritis, whereas lower strains were not sufficient to induce anti-inflammatory actions.

## Discussion

The systematic investigation of cellular responses to mechanical signals requires well characterized and reproducible methods. *In vitro* cell stretching instruments encompass the possibility to strain cells in monolayer cyclically in a controlled and defined manner by deforming the substrate where the cells were attached. The system is well investigated and established [15,19,81] but nonetheless requires some considerations. It has been reported that not the complete membrane strain is transferred to the cells attached on it. Measured in direction of the strain, in uniaxial experiments 79% ± 34% of the strain were transferred to fibroblasts [82] and 63% ± 11% were transferred to tenocytes [83]. In other experiments, 37% ± 8% and 45–60% of biaxial strains were transferred to tenocytes and bone marrow-derived stromal cells [15,83]. Gilchrist et al. (2007) pointed out that some cells exhibit extremely different strain behavior to the applied load. This may be traced back to alignment of cells relative to the direction of the load as well as cell size and shape, number of adhesion sites and organization of stress fibers within the cytoskeleton [15,82]. These factors are among others also determined by cell density. Furthermore, several studies showed that only within the central area of the wells strains were homogeneous [15,84]. Moreover, when the deformable culture surfaces were pulled over circular loading posts, biaxial strain was observed only at the center of the membranes. At the outer parts, where the membrane is pulled over the edges of the loading posts, cells experience uniaxial strains. Furthermore, dynamic stimulation involves the motion of the culture substrates and thereby fluid flow of the overlying liquid nutrient medium [85]. This leads to shear stresses that act on the cells and this might influence the mechanically induced outcome. Bieler et al. (2009) published a full-field mechanical characterization of the strain distribution within the deformable membranes. They observed that in cyclic tensile measurements, with an increasing number of cycles, the membranes did not behave consistently. The measured membrane strain was higher than the mean strain reported by the controller at all analyzed cycle numbers. This offset increased with the number of cycles applied, maybe due to changes in the material properties of the membranes during repeated use [15]. Thus, not only cell structure, cell shape, and cell orientation but also the position and attachment of the cells on the culture surface influence the real achieved strain. Pooling the responses of individual cells in a heterogeneous population could lead to misinterpretation of the data. To overcome this shortcoming, staining of individual cells could be more accurate. The distribution of different strains on the culture plate might correlate with the response.

The transfer of results from two-dimensional loading to three-dimensional and/or *in vivo* conditions remains questionable. It is a clear limitation of this method that cells are strained in monolayer where only one surface is elongated. *In vivo* chondrocytes are rounded in shape and surrounded by a matrix in normal cartilage, wherefore strains apply at all sides of the cell membrane. Additionally, in most cartilaginous tissues, the number of cell-cell contacts is limited, whereas in the reviewed studies, cells were mostly cultured until confluence. Methods with three-dimensional loading conditions might overcome this limitation. These use cartilage plugs or cell-seeded scaffolds to provide more physiological loading conditions. In this context, mechanical loading has become a promising stimulus to optimize cartilage tissue engineering [7,86]. However, the outcome depends largely on the loading parameters used [86]. Kock et al. (2012) pointed out in their review that “it is necessary to investigate which specific (combinations of) mechanical stimuli result in optimal response of the cells” [86]. Here, research on cartilage adaptation to mechanical loading that is needed to improve growth and mechanical properties of tissue engineered cartilage, might benefit from two-dimensional experiments. This is because the loading characteristics (strain magnitude, loading frequency, loading duration, and waveform) can be configured and controlled easily [16]. It is one advantage against three-dimensional designs that the load input at the cell can be quantified more precisely because the membrane strain is directly transferred to the attached cell. During loading of three-dimensional constructs, the cells are imbedded into an extracellular matrix and therefore exposed to different types of loading (strain, compression, shear, hydrostatic pressure). Furthermore, due to the mechanical properties of the surrounding matrix, it is not clear which specific mechanical signals are sensed by the cell.

Due to the ease of use of the two-dimensional design, various loading protocols can be tested and might provide a basis for loading protocols for the more complex three-dimensional methods. Two-dimensional designs should be used to identify fundamental relationships between loading protocols and cellular response, whereas three-dimensional methods should be used to investigate a more general behavior of the cells in interaction with their surrounding matrix. In the future, a combination of both loading methods could effectively contribute to a better understanding of loading induced chondrocytes response. Therefore, further information is needed to understand which strain magnitude of chondrocytes in three-dimensional constructs is gained by what specific kind of loading.

When opposing the anabolic and catabolic effects of CTS on chondrocytes gained in this review, we observed the complexity of the mechanisms and responses due to studies with differing results. Furthermore, several strain magnitudes, loading frequencies and loading durations are combined which makes it difficult to determine clear thresholds between anabolic and catabolic. Nevertheless, due to the summarized facts, we suggest that in a non-inflammatory environment loading protocols up to 3% cell strain, 0.17 Hz and 2 h could be determined as “low CTS”, between 3–10% cell strain, 0.17 Hz—0.5 Hz and 2–12 h as “moderate CTS” and above 10% cell strain, 0.5 Hz and 12 h as “high CTS”. Loading duration might be the key parameter in triggering gene expression in response to CTS (Fig. 3, Table 3). In an inflammatory environment, values are different and lower.

At the protein level, results are diverging and parameters like loading frequency and culture plate coating have to be taken into consideration. Furthermore, without studying the protein synthesis and amount, changes mRNA levels have to be interpreted carefully. One has to consider that increased mRNA levels do not necessarily lead to increased protein levels. Due to the permanent remodeling of the matrix, protein levels might not change while mRNA expression increases or decreases. It would be interesting to confirm and complement these results with future studies to better describe how other ECM proteins react in response to CTS on the gene, but especially on the protein level. Furthermore, information about the localization and integrity of ECM proteins would be of interest, because these factors also affect the mechanical properties of articular cartilage.

Furthermore, the native loading condition of a cell source could affect the cellular response to mechanical loading because it has been shown that chondrocytes from differently loaded regions in cartilage have different phenotypic expressions [87]. However, among the reviewed studies there were no obvious differences between the response of chondrocytes e. g. from the temporomandibular joint [13,45,57,60] and from the knee joints [33,36]. Though, an experiment investigating this effect within one experiment is missing. There were two studies that were excluded from the review because in these studies human articular chondrocytes from patients that underwent knee joint replacement surgery have been used [88,89]. From the information in these publications, we could not exclude that these cells had already undergone changes for osteoarthritis. It is reported by earlier studies that cells from arthritic cartilage show an altered expression of ECM proteins than cells from healthy tissue [90]. Indeed, one of the above mentioned studies showed an increased mRNA expression in chondrocytes from osteoarthritic tissue in response to a long-lasting CTS (24 h) [88], whereas cells from healthy tissue decreased mRNA expression at the same loading protocol [33,36]. The other study [89] reported that aggrecan mRNA levels were down-regulated when cells were loaded for 2 h a day (at three consecutive days) with 1 h rest in between 0.5% or 3% strain [89]. Chondrocytes from healthy tissue, however, did only down-regulate aggrecan mRNA when CTS lasted longer than 16 hours continuously [13,27,33,36]. Nonetheless, more studies are needed which compare the effect of CTS on chondrocytes from healthy tissue to chondrocytes from osteoarthritic tissue. This would improve the knowledge about the role, the limitation, and the potential of loading in the therapy of osteoarthritis.

It is surprising that the protocols in so many of the reviewed publications continued up to 96 h without interruptions. In 46 experiments within the reviewed publications, chondrocytes were stretched longer than 16 h continuously. Physiologically, repeated loading with interruptions occur in daily life. Only Perera et al. (2010) used an interrupted loading protocol (90 min a day on two following days) [32]. More of those physiological relevant loading durations should be investigated, so that the results from *in vitro* studies can be better transferred to and compared with *in vivo* conditions.

The threshold of strain magnitude between anabolic and catabolic actions of normal chondrocytes could lie at about 10–12% strain. Above this value, mainly catabolic responses were observed, whereas below, mostly anabolic actions occurred. Comparing this value with *in vivo* conditions is not easy because the *in vivo* deformation of cells during physiological loading of a human joint is not well known. However, it has been shown that during cartilage compression, the cell´s height (in split line direction) is reduced whereas the cell´s width (perpendicular to the split line) is increased [91–93]. From the following considerations, one can assume that under physiological cartilage loading this increase in width represents a cell elongation of about 5%: It is estimated that a peak hydrostatic pressure of 3.45 MPa occurs in the femoral cartilage during a squat [94]. Consequently, Herberhold et al. (1999) showed in a cadaver experiment that loading an intact human knee joint with similar stresses (peak pressures of around 3.6 MPa) lead to 30 ± 10% mean reduction of the initial femoral cartilage thickness after 214 minutes of static loading. Guilak et al. (1995) in turn applied 19% compressive strain to the superficial zone of full-depth explants of articular cartilage and subchondral bone. With these strains chondrocytes in this zone experienced a significant compression-induced radial expansion of 4.7 ± 4.1% after 20 minutes [92]. Madden et al. (2013) showed *in vivo* that 10–20% compressive strain of cartilage led to about 5–13% cell width strain, depending on the area the cells were located [12]. Similarly, it has recently been calculated that the Green-Lagrange strain for cell width increased by 0.17 ± 0.02 when bovine cartilage tissue was compressed with a 15% nominal tissue strain [91]. Extreme tissue strains of 80% cartilage compression increased the cell strain only by an additional 2–3% [95,96]. This suggests that chondrocytes *in vivo* are not subjected to more than 15% cell elongation. However, one has to consider that chondrocytes come from different layers within articular cartilage. Within these layers cells experience different physical forces and may therefore respond differently to the same strain magnitudes. Nonetheless, as showed in two of the reviewed papers, chondrocytes might also tolerate higher strains (20%; 24%) without inducing catabolic actions, although these strains might rather be un-physiologic [14,52].

There is a fine balance between anabolic and catabolic actions in chondrocytes in response to CTS. We suggest that in a non-inflammatory environment loading protocols below 3% strain, 0.17 Hz and 2 h result in weak or no biological responses. Loading protocols between 3–10% strain, 0.17 Hz—0.5 Hz and 2–12 h tend to induce anabolic responses, whereas above 10% strain, 0.5 Hz and 12 h, catabolic events occur. However, this review not only shows that each of the three loading parameters (magnitude, frequency, duration) but also that the environment of the cell contributes to the shift towards either anabolic or catabolic actions.

To provide better comparability of studies and better transition to three-dimensional conditions, we suggest considering the following hints in the future: Loading conditions should be as physiological as possible and should include pauses. Therefore, we advise to apply loading frequencies between 0.5 and 2.5 Hz, loading magnitudes between 0.5 and 15%, and loading durations shorter than 12 h. Further, culture plates should be uncoated or coated with the cartilage-specific collagen II. Data should be collected not only immediately after the last loading cycle but also after a recovery time. All parameters that could affect the cellular outcome should be explained in details.

## Conclusion

Results from *in vitro* experiments with CTS disclose further information about the effect of mechanical signals on the biological response of chondrocytes. Many factors are involved in the synthesis and remodeling of the ECM in response to loading. It is important to look not only at single isolated parameters and to combine information from different studies. A better understanding of the relationship between specific loading parameters and chondrocyte response will be useful for the development of tissue engineered cartilage. Furthermore, the simulation of an inflammatory environment allows new insights into the anabolic capabilities of specific loading protocols in rehabilitation and therapy of degenerative joint disease like osteoarthritis.

## Supporting Information

S1 Checklist PRISMA checklist. (PDF)
